# Current Trends and Perspectives in the Application of Polymeric Materials to Wastewater Treatment

**DOI:** 10.3390/polym13071089

**Published:** 2021-03-30

**Authors:** Ricardo N. Coimbra, Marta Otero

**Affiliations:** 1 Department of Environment and Planning, University of Aveiro, Campus Universitário de Santiago, 3810-193 Aveiro, Portugal; ricardo.coimbra@ua.pt; 2 Centre for Environmental and Marine Studies (CESAM), University of Aveiro, Campus Universitário de Santiago, 3810-193 Aveiro, Portugal

Water with the necessary quality is indispensable to the functioning of most of the known life forms, being essential to human health, social and economic development, and ecosystems functioning. However, only 2.5% of all water on Earth is freshwater, and less than 1% is accessible, its availability being actually affected by climate change and direct human impacts. Furthermore, population growth and industry expansion have been leading to the continuous degradation of freshwater quality around the world. Under these circumstances, ensuring sufficient and safe water supplies for everyone is one of the Sustainable Development Goals (SDGs) set by the United Nations General Assembly in 2015 for the year 2030. For this goal to be achieved, the development and implementation of appropriate and efficient wastewater treatments is a major challenge.

The application of polymers and polymeric materials in wastewater treatment is a research field that has greatly developed from the end of the last century. The very nature, structure, and versatility of polymers make them useful for many applications, including wastewater treatment processes. Conventional and novel approaches have been elaborated or refined by researchers from different areas, who have demonstrated that polymers and polymeric materials may have an important role not only in the removal of pollutants of different origin and nature from wastewater but also in the recycling of materials and the improvement of wastewater efficiency and economy.

In view of the relevant contribution of polymers and polymeric materials to the conservation of the aquatic environment, namely by their application in wastewater treatment, this Special Issue (SI) was launched for the publication of original research or review papers within this topic. The aim was to bring forth the challenges and discuss current trends and perspectives in the utilization of polymers and polymeric materials—either synthetic or natural—for the treatment or purification of wastewater.

Eleven research works [1,2,3,4,5,6,7,8,9,10,11] by distinguished international authors were published within this SI on “Current Trends and Perspectives in the Application of Polymeric Materials for Wastewater Treatment”, which covered a wide range of issues related to this topic.

Abdulsalam et al. [1] developed hydrophilic hybrid polyvinylidene fluoride (PVDF)-polyethylene glycol (PEG) ultrafiltration membranes loaded with Nano-MgO (NMO) by using a phase inversion technique. These authors demonstrated that NMO incorporation not only improved the membranes’ antifouling properties and permeation performance but also allowed for an enhanced color separation from palm oil mill effluent, which was related to the synergism between surface deprotonation and pore size screening. For their part, Linhares et al. [6] loaded silver nanoparticles (AgNPs) in microfiltration polymeric membranes (15 wt.% polyethersulfone and 7.5 wt.% polyvinylpyrrolidone in *N*,*N*-dimethylacetamide) by the sputtering technique. They demonstrated the efficiency of this technique to load AgNPs, which provided membranes with biocidal properties resistant to biofouling and made them proficient for water disinfection treatments.

Mia et al. [8] loaded iron on waste silk fibers previously grafted with polydopamine by oxidative polymerization to produce wSF-DA/Fe, which was tested for the catalytic removal of toxic dyes (Methylene Blue, Cationic Violet X-5BLN, and Reactive Orange GRN). The authors postulated that the dye removal was due to the synergistic effect of free radicals and reactive species, which resulted from a heterogeneous Fenton reaction and oxidized the dyes into colorless nontoxic substances. The catalytic performance of wSF-DA/Fe was affected by the H_2_O_2_ concentration, initial dye concentration, temperature, and presence of electrolytes (NaCl, Na_2_SO_4_). Aiming at the catalytic removal of Rhodamine B (RB) dye, Ansari et al. [2] synthesized Fe_3_O_4_ nanoparticles (NPs) and sodium dodecyl sulfate (SDS) coated Fe_3_O_4_ NPs (SDS@Fe_3_O_4_) by the co-precipitation method. RB degradation was tested in presence of H_2_O_2_, H_2_O_2_ and Fe_3_O_4_ NPs, and H_2_O_2_ and SDS@Fe_3_O_4_ NPs, the latter providing an increased catalytic removal, as the SDS coating avoided the aggregation of Fe_3_O_4_ and the associated efficiency reduction.

In the field of adsorptive treatments, Khan et al. [5] investigated RB adsorption by a novel solvent impregnated resin (SIR). SIR, which was produced by modifying the cationic polymeric resin Dowex 5WX8 with the solvent t-butyl phosphate, adsorbed RB mainly by electrostatic interactions and π–π bonding. The authors optimized the operational conditions, viz. pH, SIR dosage, and contact time, for the adsorptive removal of RB. The adsorptive removal of Cr(VI) from water was studied by Yang et al. [11], who produced for this purpose activated carbon microspheres (SLACM), using sodium lignosulfonate ((SL), a waste from the pulp and paper industry) as raw material. The synthesis of SLACM, which was to be attained with no binder addition, consisted of (i) amination of chloromethylstyrene-divinylbenzene-styrene copolymer (CMPS) with 1,3-diaminopropane; (ii) Mannich reaction of SL and amino CMPS to produce the adsorbent resin microsphere; (iii) impregnation with ZnCl_2_ and pyrolysis at 600 °C. The adsorption of Cr (VI) by the produced SLACM was shown to be favored by decreasing pH (within pH 2 and 9) and increasing temperature (within 20 and 40 °C). Hoshima et al. [4] aimed at the adsorption of the rare earths dysprosium (Dy) and neodymium (Nd) from water as a previous necessary step for their subsequent recovery. With this objective, the authors produced a new adsorbent by the radiation-induced graft polymerization of methacrylate with a long alkyl chain on a PE/PP nonwoven fabric and the subsequent loading of 2-ethylhexyl hydrogen-2-ethylhexylphosphonate by hydrophobic interaction and chain entanglement between the alkyl chains. Four different methacrylate monomers were tested, namely butyl methacrylate (BMA), hexyl methacrylate (HMA), dodecyl methacrylate (DMA), and octadecyl methacrylate (OMA). Only the OMA-adsorbent was stable under subsequent uses, which was related to the suppression of EHEP losses due to the strong hydrophobic interaction and chain entanglement between the long alkyl chains. Moreover, OMA-adsorbent was efficient in the adsorption of Dy (III) and Nd (III) from water, being a promissory material for the recovery of rare-earth metals from NdFeB permanent magnet scraps.

Four papers in the SI dealt with the adsorptive removal of pharmaceuticals [3,7,9,10]. Mashile et al. [7] synthesized a magnetic mesoporous carbon/-cyclodextrin–chitosan (MMPC/Cyc-Chit) nanocomposite for the adsorption of fluoroquinolones (FQs), viz. danofloxacin, enrofloxacin, and levofloxacin, from different water samples, including a synthetic mixture of the FQs, the influent and effluent from a wastewater treatment plant, river water, and tap water. The authors found that incorporating biodegradable polymers such as chitosan and cyclodextrin into magnetic mesoporous carbon brought about a nanocomposite with large surface area and adsorption capacity. This was a very complete study, which included the optimization of operational parameters (pH, mass of MMPC/Cyc-Chit, and sonication power) by a response surface methodology, kinetic and equilibrium modeling, assessment of regeneration and reusability, thermodynamic parameters determination, and cost analysis for the synthesis of MMPC/Cyc-Chit. Mohammadi et al. [9] synthesized a poly(styrene-block-acrylic acid) diblock copolymer/Fe_3_O_4_ magnetic nanocomposite (P(St-b-AAc)/Fe_3_O_4_)) and tested it for the adsorption of antibiotic ciprofloxacin from synthetic wastewater. This work included the optimization of operational parameters, namely antibiotic concentration, pH, nanocomposite mass, and contact time, and showed that P(St-b-AAc)/Fe_3_O_4_ was efficient in the adsorptive removal of ciprofloxacin. Aiming at the adsorption of selective serotonin reuptake inhibitor (SSRI) antidepressants and their metabolites from water, Gornik et al. [3] optimized the synthesis of molecularly imprinted polymer (MIP) adsorbents in which the SSRI sertraline was used as template. Different MIPs were synthesized by varying the functional monomer, the porogen, and/or the template form, which were shown to largely affect the adsorbent performance of the resulting material, so the authors selected the most efficient. Even when the selected MIPs had a relatively lower surface area than conventional activated carbons, they displayed a larger adsorption capacity in real wastewater samples. Apart from the MIPs optimization and characterization, this work included the assessment of their reusability, occurrence of cross-reactivity, adsorption kinetics, matrix effects, upscale, and leaching, with authors pointing out the necessity of carrying out future work at a larger scale to confirm the advantages of the synthetized materials. In order to overcome the drawbacks of MIPs, mainly the small number of recognition sites per unit of volume and the low mass transfer, surface molecular imprinting on magnetic yeast (MY) was carried out by Qiu et al. [10], who synthesized highly selective magnetic yeast-molecularly imprinted polymers (MY@MIPs) for the adsorptive removal of antibiotic sulfamethoxazole (SMX). For the production of MY@MIPs, these authors started by preparing nano-Fe_3_O_4_ by an in situ one-step procedure, which was loaded onto yeast cells to obtain MY. Then, MY was used as core for the polymerization of MIPs using SMX as template to produce MY@MIPs. The authors compared MY@MIPs with MY and non-imprinted MY@NIPs (synthesized in the same way as MY@MIPs but in the absence of template) and evidenced the superior SMX adsorption capacity of MY@MIPs. Furthermore, besides the selectivity of MY@MIPs towards SMX in the presence of other pharmaceuticals and in real wastewater, the reutilization capability of this material was also proved, pointing to its possible application as an alternative adsorbent for SMX selective removal from wastewater.

Membrane filtration and catalytic applications together with the utilization of polymeric materials for the adsorptive removal of pollutants were discussed in this SI. Furthermore, it is worth highlighting the attention given to the adsorptive removal of pharmaceuticals from wastewater. Since the 1990s, a growing scientific concern about pharmaceuticals may be inferred from the number of related publications, and this SI was no exception. Such a concern is mainly related to (i) the analytic development that has made possible their detection at trace levels and the confirmation of their ubiquity in environment; and (ii) the ecological risks related to their potential to cause physiological responses in non-target individuals, including endocrine disruption and antibiotic resistance. An important benefit of pharmaceuticals removal by adsorption is that such a treatment does not result into the formation of by-products, which in some cases can be more hazardous than the parent compounds. 

Polymers have been used for long in conventional wastewater treatment for the flocculation/coagulation of solids, so that they may be easily separated from water. However, the SI hereby presented makes evident that polymers and polymeric materials may have many new and varied applications in wastewater treatment. Most published works take advantage of polymers’ versatility and capacity to be combined or modified to produce advanced materials with relevant features for water treatment. In fact, polymer modification is presently a hot topic, since it allows for the development of specific and even smart materials for target applications in different sectors, including wastewater treatment. Owing to the efforts of polymer engineers and scientists, many alternative and advanced polymeric materials are continuously developed. In the specific case of wastewater treatment, polymer applications have become very important due to the increased pollutant removal efficiencies that they offer, so this is a research field in great expansion. The progress in the last years and the successful applications of polymer and polymeric materials are reflected by the high quality works published within this SI.

## Data Availability

Data sharing is not applicable to this article.
